# MRI grading for the prediction of prostate cancer aggressiveness

**DOI:** 10.1007/s00330-021-08332-8

**Published:** 2021-11-08

**Authors:** M. Boschheidgen, L. Schimmöller, C. Arsov, F. Ziayee, J. Morawitz, B. Valentin, K. L. Radke, M. Giessing, I. Esposito, P. Albers, G. Antoch, T. Ullrich

**Affiliations:** 1 Department of Diagnostic and Interventional Radiology, Medical Faculty, University Dusseldorf, Moorenstr. 5, 40225 Düsseldorf, Germany; 2 Department of Urology, Medical Faculty, University Dusseldorf, 40225 Düsseldorf, Germany; 3 Department of Pathology, Medical Faculty, University Dusseldorf, 40225 Düsseldorf, Germany

**Keywords:** Prostatic neoplasms, Multiparametric magnetic resonance imaging, Diffusion magnetic resonance imaging, Neoplasm grading, Magnetic resonance imaging, interventional

## Abstract

**Objectives:**

T o evaluate the value of multiparametric MRI (mpMRI) for the prediction of prostate cancer (PCA) aggressiveness.

**Methods:**

In this single center cohort study, consecutive patients with histologically confirmed PCA were retrospectively enrolled. Four different ISUP grade groups (1, 2, 3, 4–5) were defined and fifty patients per group were included. Several clinical (age, PSA, PSAD, percentage of PCA infiltration) and mpMRI parameters (ADC value, signal increase on high b-value images, diameter, extraprostatic extension [EPE], cross-zonal growth) were evaluated and correlated within the four groups. Based on combined descriptors, MRI grading groups (mG1–mG3) were defined to predict PCA aggressiveness.

**Results:**

In total, 200 patients (mean age 68 years, median PSA value 8.1 ng/ml) were analyzed. Between the four groups, statistically significant differences could be shown for age, PSA, PSAD, and for MRI parameters cross-zonal growth, high b-value signal increase, EPE, and ADC (*p* < 0.01). All examined parameters revealed a significant correlation with the histopathologic biopsy ISUP grade groups (*p* < 0.01), except PCA diameter (*p* = 0.09). A mixed linear model demonstrated the strongest prediction of the respective ISUP grade group for the MRI grading system (*p* < 0.01) compared to single parameters.

**Conclusions:**

MpMRI yields relevant pre-biopsy information about PCA aggressiveness. A combination of quantitative and qualitative parameters (MRI grading groups) provided the best prediction of the biopsy ISUP grade group and may improve clinical pathway and treatment planning, adding useful information beyond PI-RADS assessment category. Due to the high prevalence of higher grade PCA in patients within mG3, an early re-biopsy seems indicated in cases of negative or post-biopsy low-grade PCA.

**Key Points:**

• *MpMRI yields relevant pre-biopsy information about prostate cancer aggressiveness.*

• *MRI grading in addition to PI-RADS classification seems to be helpful for a size independent early prediction of clinically significant PCA.*

• *MRI grading groups may help urologists in clinical pathway and treatment planning, especially when to consider an early re-biopsy.*

**Supplementary Information:**

The online version contains supplementary material available at 10.1007/s00330-021-08332-8.

## Introduction

The combination of clinical parameters such as PSA testing and multiparametric magnetic resonance imaging (mpMRI) of the prostate using PI-RADS (Prostate Imaging–Reporting and Data System) is highly sensitive in prostate cancer (PCA) detection of not only large aggressive PCA but also small, early-stage and/or low-risk PCA in the clinical routine. Although PI-RADS is designed for assessing the likelihood of clinically significant PCA (ISUP ≥ 2) being present, especially when using a low threshold for biopsy, e.g. patients in PI-RADS overall assessment category 3, and/or if patients receive additional systematic biopsies, the rate of low-grade, non-significant PCA (ISUP 1) increases as high as 90% of all diagnosed PCA [1, 2]. Since there are many different therapy options for PCA, MRI can provide helpful assistance for treatment decisions, as established in the EAU guidelines. Whereas aggressive PCA demands urgent, extensive treatment, i.e. radical resection of the prostate, including the seminal vesicles and the pelvic lymph nodes, other therapy options are available for lower risk PCA [3, 4]. More invasive procedures have certain risks and may entail complications like incontinence or erectile dysfunction [5]. In cases of less aggressive PCA, nerve-sparing surgery and radiotherapy, including brachytherapy or other focal therapies, can be an option, among others. For PCA within ISUP 1 (in studies ISUP 2), active surveillance is also a possible strategy. Since serial follow-up mpMRI is part of this strategy, it is necessary to evaluate and define more specific imaging descriptors indicating PCA aggressiveness and PCA progress so that re-biopsy may be avoided or delayed in stable cases [6].

Different risk calculators are available to predict initial PCA aggressiveness and the risk of possible PCA progression, including parameters like Gleason score, PSA value, or demographic aspects [7, 8]. There is evidence that mpMRI is also helpful in estimating the initial PCA aggressiveness and supporting the clinical decision-making process [9–13]. Studies revealed that ADC values are negatively correlated with the Gleason score [14–16]. Additionally, it has been shown that the combination of functional and anatomic MRI sequences cannot only differentiate between cancerous areas and benign prostate tissue but also clearly define PCA margins so that tumour size and potential extraprostatic extensions (EPE) can be assessed. However, as of now, there is no consensus of MRI-based prediction of PCA aggressiveness [17].

The aim of this study is the systematic evaluation of clinical and MRI parameters for the prediction of PCA aggressiveness in biopsies next to the PI-RADS evaluation and suggestion of an MRI-based grading system to assist in the choice of the appropriate therapy regime.

## Materials and methods

### Study design

The local ethics committee approved the study (Medical Faculty of the Heinrich-Heine-University Düsseldorf; Study-ID: 2,017,034,171). Written informed consent was obtained from every patient. Four groups (biopsy ISUP grade group 1, 2, 3, and 4 combined with 5) were defined and 50 consecutive patients per group were included to ensure equal group size. All patients received mpMRI of the prostate at our institution between January 2016 and March 2020. Subsequently, targeted MRI/ultrasound fusion-guided biopsy combined with systematic 12-core transrectal ultrasound-guided prostate biopsy was conducted. Only patients with biopsy-confirmed PCA were included. None of the patients had a known prostate cancer. Exclusion criteria were previous treatment for prostate cancer and incomplete or non-diagnostic MRI. Clinical and MRI parameters were defined and retrospectively correlated with different biopsy ISUP grade groups. Clinical information contained age, PSA, PSAD, and percentage of infiltration in biopsy. MRI parameters included ADC values of PCA lesions, PCA diameter, EPE, cross-zonal growth, and signal increase on high b-value images. The primary study endpoint was to prove if there are significant differences in the mentioned parameters between the histopathologic biopsy ISUP grade groups. Secondary objective was the biopsy ISUP grade group correlation with defined MRI grading groups (mG1 to mG3), based on the combination of different descriptors.

### Imaging acquisition

All mpMRI scans were conducted on 3-T MRI scanners (Magnetom TIM Trio: *n* = 164, Prisma: *n* = 61, or Skyra: *n* = 23; Siemens Healthineers) using either an 18-channel phased-array surface coil combined with a 32-channel spine coil or a 60-channel phased-array surface coil. MRI parameters were chosen according to international recommendations and contained T2-weighted turbo spin echo (TSE) sequences in 3 planes (T2WI; axial: voxel size 0.5 × 0.5 × 3.0 mm; FOV 130 mm), diffusion-weighted imaging (DWI; ss-EPI [single-shot EPI DWI] and rs-EPI [readout-segmented multi-shot EPI DWI, RESOLVE; Siemens Healthineers]; voxel size 1.4 × 1.4 × 3.0 mm; b-values 0, 500, 1000 s/mm^2^ plus calculated 1800s/mm^2^), and dynamic contrast-enhanced imaging (DCE; T1 vibe; voxel size 0.8–1.5 × 0.8–1.5 × 3.0 mm, scan time 3 min, temporal resolution 7 s) [18]. Apparent diffusion coefficient (ADC) parameter maps were calculated by the scanner using the standard monoexponential model. Ss-EPI was acquired in 125 and rs-EPI in 96 of 200 patients. Further details of acquisition parameters are provided in the supplementary data (Supp. Tables 2–4).

### Biopsy

Targeted MRI/ultrasound fusion-guided biopsy and subsequent systematic 12-core TRUS-GB were conducted on an MRI/US fusion-guided biopsy system with elastic registration (UroNAV, Invivo) using an 18-G fully automatic biopsy gun (Bard Medical) by experienced urologists with over 5 years of experience. Two targeted cores were taken from each lesion.

### Image analysis

MpMRI data were retrospectively evaluated by three readers in consensus (M.B., T.U., and L.S.) with 3, 7, and 10 years of experience. By the time of the imaging analysis, the readers were blinded towards the histopathologic ISUP groups. Prostate volume was measured by software volumetric (DynaCAD, Philips Healthcare) and PSA density (PSAD) was calculated by dividing PSA blood levels by prostate volume. First, it was evaluated if a PCA index lesion (IL) was visible on mpMRI, defined as PIRADS (v2.1) assessment category 3, 4, or 5. Only one IL was assessed per patient. If there was more than one lesion, the one with the highest PI-RADS v2.1 assessment category or the one with EPE, if present, or the largest lesion was chosen. For all visible PCA IL, maximum diameter was measured in T2w sequences. EPE or seminal vesicle infiltration (cT3 stage) was present if PCA crossed the prostate pseudocapsule (≥ 3 mm) or extended per continuitatem into the seminal vesicles. Cross-zonal growth was defined as growth in the peripheral zone (PZ) and expansion into the transition zone (TZ) or vice versa indicating invasive behaviour. In DWI, lesions were classified as positive if they were visible and different from the background in high b-values (calculated b1800). ADC values were measured by placing a circular region of interest (ROI) into the visually darkest PCA area. The PCA were assigned to defined MRI grading groups (mG1 to mG3) containing quantitative and qualitative information of patients and PCA lesions (Table 1). The definition of the different groups was based on previous studies showing that ADC values using a threshold from 750 to 900 µm^2^/s may help to estimate lesion aggressiveness and on clinical experience [16, 19, 20].

**Table 1:** Definition of MRI grading groups to assess the PCA aggressiveness

		MRI grading group		
		mG1	mG2	mG3
DWI	Hypointense on ADC	> 900 (rs-EPI) > 800 (ss-EPI)	< 1000 (rs-EPI) < 900 (ss-EPI)	< 900 (rs-EPI) < 800 (ss-EPI)
	Hyperintense on high b-value images	0 or 1	0 or 1	1
		and	and/or	and
T2	Focal suspicious signal decrease	Discreet	Discreet, overlayed, or clear	Clear
	Multifocal or cross-zonal growth	0	0 or 1	0 or 1
		and	and	or
EPE		0	0	1

*PCA*, prostate cancer; *T2*, T2-weighted imaging; *DWI*, diffusion-weighted imaging; *ADC*, apparent diffusion coefficient; *MG*, MRI grading group; *rs-EPI*, readout-segmented multi-shot echoplanar imaging; *ss-EPI*, single slice echoplanar imaging; *EPE*, extraprostatic extension

### Statistical analysis

Statistics were performed using IBM SPSS® Statistics (Version 27, IBM Corp). *p* values < 0.05 (marked in bold) were defined as statistically significant. Bonferroni-corrected analysis of variance (ANOVA) was used to compare clinical and mpMRI parameters between different ISUP grade groups. For correlation analyses, the Kendall Tau correlation coefficient *τ* was calculated. Correlation strengths were graded as suggested by Cohen: small (< 0.3), moderate (0.3–0.5), and large (> 0.5) [21]. For the prediction of ISUP group based on MRI images, a multivariable statistical analysis was performed using a linear mixed model (LMM). The performance of combinations of parameters in discriminating between different ISUP grade groups was analyzed using restricted maximum likelihood (REML) to account for within-patient correlations. Models for rs-EPI and ss-EPI were calculated, respectively.

## Results

### Patients

Of the entire patient cohort of 200 patients (mean age 68 ± 8 years; median PSA 9.3 ng/ml, IQR 6.2–12 ng/ml; median PSAD 0.21 ng/ml/cm^3^, IQR 0.15–0.33 ng/ml/cm^3^), 50 patients had PCA classified as biopsy ISUP 1, 50 patients ISUP 2, 50 patients ISUP 3, and 50 patients ISUP 4 or 5.

### Comparison of clinical and MRI parameters among biopsy ISUP grade groups

In 187 of all 200 patients, a PCA suspicious IL was found on mpMRI. In 13 patients, PCA was indistinct or masked. In ISUP 1, only 40/50 of the PCA were visible in mpMRI, whereas all PCA were visible in ISUP 4 and 5. 39/50 of the IL in ISUP 1 were localized in the peripheral zone, 35/50 in ISUP 2, 39/50 in ISUP 3, and 39/50 in ISUP 4 and 5. 7/50 of the IL in ISUP 1 were localized in the transition zone (ISUP 2: 9/50; ISUP 3: 9/50; ISUP 4–5: 7/50), and 4/50 of the IL in ISUP 1 were localized in the anterior stroma (ISUP 2: 6/50; ISUP 3: 2/50; ISUP 4–5: 4/50). Except for PCA diameter (*p* = 0.092), ANOVA analysis showed significant differences for the means of all examined clinical and MRI-based parameters among the different ISUP grade groups with *p* = 0.004 for signal increase on high b-value images, *p* = 0.044 for ADC values using ss-EPI (*n* = 125 patients), and *p* < 0.001 for all others, respectively (Table 2).

**Table 2:** Comparison of clinical and MRI parameters of patients with different biopsy ISUP grade groups

			ISUP grade group				
			1	2	3	4–5	*p* value*
Clinical	Patients (*n*)		50	50	50	50	
	Age in years; mean ± SD		65 ± 9	67 ± 9	70 ± 8	71 ± 8	**< 0.001**
	PSA in ng/ml; median (IQR)		7.0 (5.3–9.5)	9.0 (6.4–12.8)	8.0 (6.3–11.1)	11.6 (6.6–15.2)	**< 0.001**
	PSAD in ng/ml/cm^3^; median (IQR)		0.16 (0.11–0.22)	0.23 (0.13–0.37)	0.20 (0.13–0.30)	0.26 (0.20–0.41)	**< 0.001**
	Percentage of infiltration in biopsy; median (IQR)		15 (5–40)	60 (35–80)	50 (25–80)	60 (25–80)	**< 0.001**
MRI	PCA visible in %		80	94	98	100	**< 0.001**
	T2 or DCE	PCA diameter in mm; median (IQR)	12 (10–14)	14 (13–18)	14 (12–16)	14 (11–18)	0.092
		Cross-zonal growth in %	10	32	44	60	**< 0.001**
		cT3 stage in %	5	9	18	38	** < 0.001**
	DWI	Signal increase on high b-value images in %	60	74	84	90	**0.004**
		ADC value (rs-EPI) in × 10^−3 mm2/s^; median (IQR)**	978 (793–1141)	959 (848–1024)	770 (707–932)	704 (608–824)	**< 0.001**
		ADC value (ss-EPI) in × 10^−3 mm2/s^; median (IQR)**	747 (599–873)	714 (652–849)	691 (495–788)	684 (581–798)	**0.044**

^*^Bonferroni-corrected multifactorial ANOVA was used to check for statistical significance

^**^Calculated for *n* = 96 (rs-EPI) and *n* = 125 (ss-EPI)

*PCA*, prostate cancer; *PSA*, prostate-specific antigen; *PSAD*, prostate-specific antigen density; *DWI*, diffusion-weighted imaging; *ADC*, apparent diffusion coefficient; *rs-EPI*, readout-segmented multi-shot echoplanar imaging; *ss-EPI*, single slice echoplanar imaging; *DCE*, dynamic contrast enhancement; *IQR*, interquartile range

*p* values < 0.05 (marked in bold) were defined as statistically significant

### MRI grading groups

**Fig. 1:**
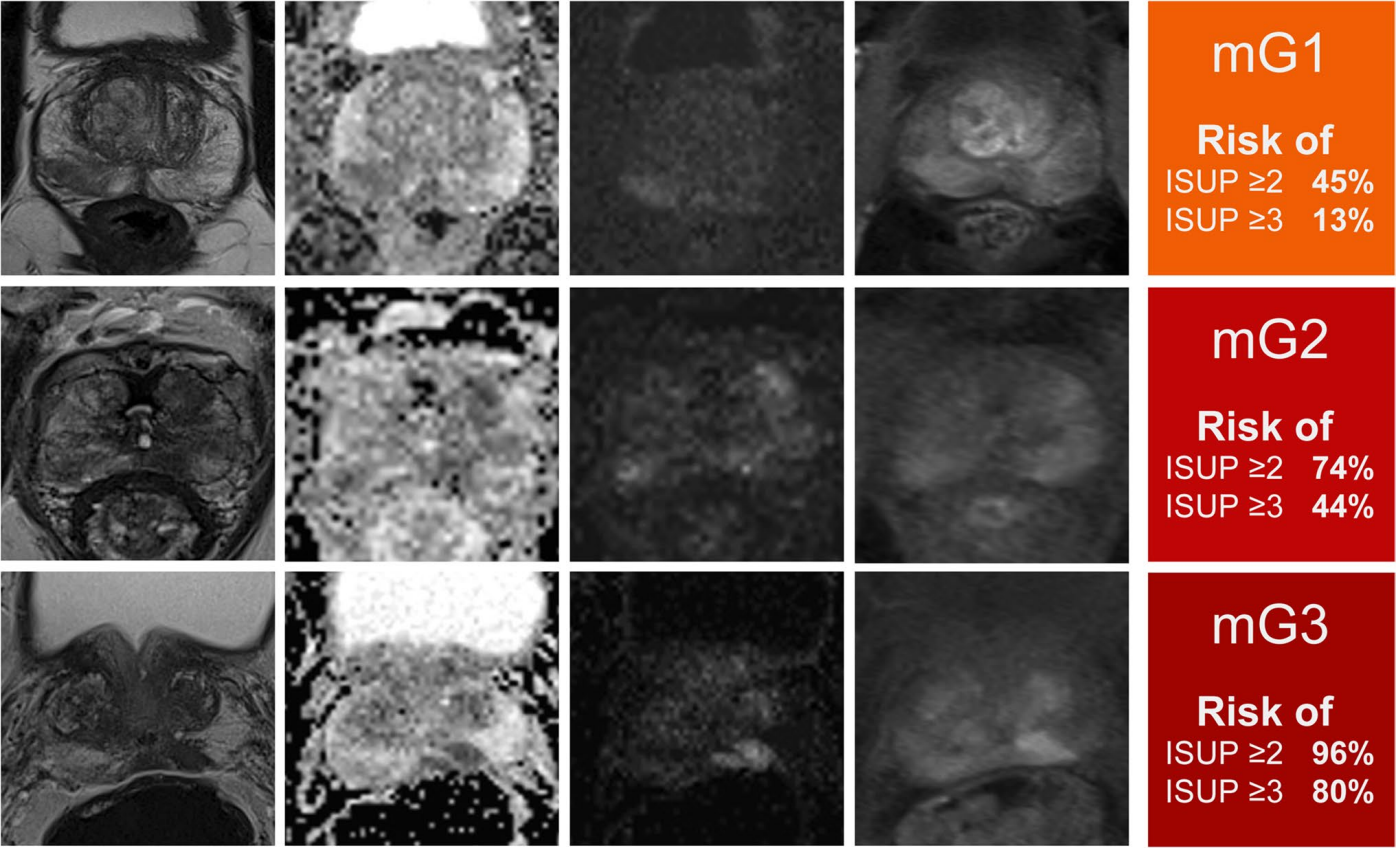
MRI grading groups 1 to 3 and estimated risk of higher grade prostate cancer with examples of mpMRI. The first column shows T2w sequences, second column ADC-maps (rs-EPI), third column high b-value DWI (b1800), fourth column DCE, and last column the risk of ISUP grade group ≥ 2 or ≥ 3 PCA. In the first line of images, a representative patient within mG1 is shown and the second line presents a patient within mG2 with focally PCA and only moderate ADC value reduction (rs-EPI). The last patient in line 3 was graded within mG3 with marked reduction in ADC value, dark focal appearance on T2w images, and bright signal on high b-value DWI

Thirty-one lesions were classified as mG1 (17/50 ISUP 1; 10/50 ISUP 2, 4/50 ISUP 3, and 0/50 ISUP 4–5), 78 lesions as mG2 (20/50 ISUP 1; 24/50 ISUP 2, 20/50 ISUP 3, and 14/50 ISUP 4–5), and 78 lesions as mG3 (3/50 ISUP 1; 13/50 ISUP 2, 26/50 ISUP 3, and 36/50 ISUP 4–5) (Table 3). The risk of having a PCA with ISUP ≥ 2 was 45% in mG1, 74% in mG2, and 96% in mG3. The risk of ISUP ≥ 3 PCA was 13% in mG1, 44% in mG2, and 80% in mG3. Examples of cases of each MRI grading group are illustrated in Fig. 1.

**Table 3:** Distribution of MRI grading groups and biopsy ISUP grade group

		MRI grading group			All
		1	2	3	
ISUP grade group	1	17	20	3	40
	2	10	24	13	47
	3	4	20	26	50
	4–5	0	14	36	50
All		31	78	78	187*

^*^13 PCa were not visible on MRI (10 with ISUP 1 and 3 with ISUP 2)

### Correlation and predictive power of the ISUP grade group among clinical and MRI parameters

All clinical parameters and almost all mpMRI parameters showed a significant correlation with the PCA ISUP grade group (Table 4; Supp. Figure 1) with the strongest association for the parameters MRI grading group (*τ* = 0.534; *p* < 0.001) and ADC values (rs-EPI) (*τ* =  − 0.468; *p* < 0.001). No significant correlation was found for PCA diameter (*τ* = 0.132; *p* = 0.71). The PI-RADS scoring system also correlated significantly with the ISUP grade groups (*p* < 0.001).

**Table 4:** Spearman correlation between clinical/MRI parameters and biopsy ISUP grade group

		*r*	*p*
Clinical	Age	0.262	** < 0.001**
	PSA	0.250	**< 0.001**
	PSAD	0.292	**< 0.001**
MRI	PI-RADS	0.231	** < 0.001**
	PCA visible	0.280	**< 0.001**
	PCA diameter	0.132	0.071
	Cross-zonal growth	0.348	**< 0.001**
	cT3 stage	0.307	**< 0.001**
	Signal increase on high b-value images	0.259	**< 0.001**
	ADC value (ss-EPI)	− 0.255	**0.045**
	ADC value (rs-EPI)	− 0.468	**< 0.001**
	MRI grading group	0.534	**< 0.001**

*PCA*, prostate cancer; *PSA*, prostate-specific antigen; *PSAD*, prostate-specific antigen density; *PIRADS*, Prostate Imaging Reporting and Data System; *ADC*, apparent diffusion coefficient; *rs-EPI*, readout-segmented multi-shot echoplanar imaging; *ss-EPI*, single slice echoplanar imaging

*p *values < 0.05 (marked in bold) were defined as statistically significant

In the linear mixed model analysis, the MRI grading group was the only parameter that showed a significant effect in prediction of the ISUP grade group in direct comparison to the other MRI parameters using ADC from rs-EPI (Table 5) (*p* < 0.001) or ADC from ss-EPI (Supp. Tab. 1).

**Table 5:** Linear mixed model with ADC values of rs-EPI DWI to evaluate the prediction of the ISUP grade group

*n* = 96		*B*	95% CI	*p*
MRI	Fixed term	3.11	2.13–4.09	** < 0.001**
	Cross-zonal growth	− 0.21	− 0.68 to 0.25	0.366
	T3 stage	− 0.07	− 0.59 to 0.44	0.778
	Signal increase on high b-value images	− 0.03	− 0.62 to 0.56	0.911
	ADC value (rs-EPI)	− 0.000328	− 0.001 to 0.0016	0.616
	MRI grading group	1 = − 1.7 2 = − 1.1 3 = Reference	− 2.7 to − 0.7 − 1.6 to − 0.6 Reference	** < 0.001**

*PCA*, prostate cancer; *DWI*, diffusion-weighted imaging; *ADC*, apparent diffusion coefficient; *B*, estimation; *CI*, 95% confidence interval; *p*, *p* value; *rs-EPI*, readout-segmented multi-shot echoplanar imaging

*p* values < 0.05 (marked in bold) were defined as statistically significant

## Discussion

MpMRI can deliver important information about PCA aggressiveness [22, 23], especially using ADC values [24, 25]. In this study, we could demonstrate that clinical and MRI-based quantitative and qualitative parameters can provide comprehensive, reliable information about the PCA ISUP grade group, potentially facilitating even more individualized, suitable therapy planning. In direct comparison with the single MRI-based parameters, a defined MRI grading group system (mG1 to mG3), incorporating different MRI descriptors, revealed the strongest effect in prediction of the final biopsy ISUP grade group, which might be useful in addition to PI-RADS.

In our multivariate analysis, almost all defined clinical and MRI parameters showed significant differences between the ISUP grade groups except for PCA diameter. Lesion size plays e.g. a role for differentiating PI-RADS 4 from 5 lesions based on tumour volume calculations of prostatectomy specimens focusing the differentiation of significant and non-significant PCA [26]. However, also smaller lesions can contain high-grade cancer, especially in early detection [27]. Somehow contrary results might be likewise due to the more detailed grading with differentiation of single ISUP grade groups and/or affected by size measuring on MRI. The parameter cross-zonal growth of PCA showed a good performance in differentiating the various biopsy ISUP groups in our analysis. As the majority of cancers are localized in the PZ and infiltrative behaviour into the TZ has been reported to occur in more aggressive cancers [28], this parameter seems to be an interesting aspect in PCA characterization and grading. Besides, the crossing of anatomical borders, not only between the different intraprostatic zones but also into extraprostatic tissue, especially occurs in higher ISUP grade groups [29] and is a general measure of PCA aggressiveness. Focusing on ADC values, in ss-EPI, technical aspects could play a role for the poorer performance compared to readout-segmented multi-shot EPI (rs-EPI). In previous studies, it has been shown that advanced DWI technology, e.g. parallel transmit EPI (ptx-EPI) or rs-EPI, delivers a significantly higher ADC reduction of PCA lesions versus healthy tissue compared to standard ss-EPI DWI [18]. The PI-RADS scoring system, designed to detect PCA with an ISUP ≥ 2, also showed a positive correlation with the pathologic biopsy ISUP groups in our analysis which also served as a measure of consistency given that PI-RADS assessment category 5 lesions contain significantly more higher ISUP grade PCA compared to the other PI-RADS categories. However, in PI-RADS 4 and 5 also ISUP 1 PCA were detected [1, 2].

Head-to-head comparison of the single MRI parameters for MRI-based grading of PCA using a linear mixed model demonstrated the best performance for a combination of parameters within MRI grading groups. This seems logical as most of the MRI parameters already demonstrated significant correlation with the ISUP grade groups and incorporating as many cancer characteristics as possible should lead to the clearest results. Other parameters failed to reach the level of significance in a LMM although they showed good correlation with the ISUP grade group distribution. A possible explanation is the outshining effect of the parameter grading group in the LMM, which possibly masks the effect of other parameters in this mixed model.

The presented MRI grading group system partly uses similar imaging features of PI-RADS for its classification, but some descriptors are not part of the PI-RADS evaluation, e.g. cross zonal growth, and single features are weighed and combined differently in comparison to the PI-RADS system. However, it has to be emphasized that the two systems have a different focus. PI-RADS primarily provides a likelihood for the presence of clinically significant PCA (ISUP ≥ 2) and the MRI grading groups providing information about PCA aggressiveness. PI-RADS differentiates category 4 and 5 lesions mainly based on the largest tumour diameter under or ≥ 1.5 mm. But in clinical settings, there are also small higher grade PCA lesions with high ADC reduction and clear imaging features or large low-grade PCA lesions with mild to moderate ADC reduction and discreet imaging features. Our results confirmed no significant correlation of the tumour diameter with the ISUP grade groups. In comparison, the MRI grading group score can express the higher or less aggressiveness to complement the PI-RADS classification and can e.g. suggest early re-biopsy in mG3 or primary follow-up in mG1 cases. This might reduce an unnecessary therapy delay and potential development of metastases due to late detection of high-grade PCA.

During active surveillance, it is important to evaluate MRI features of PCA aggressiveness and development of these features over time to trigger or postpone re-biopsy or definite treatment. The PRECISE criteria provide assistance in evaluation of lesion development in follow-up scans [30]. Our results showed that the MRI grading groups correlate with the histopathologic findings and thus might be useful to observe cancer progression or PCA aggressiveness assessment in patients planned for or within AS in an objective way. In this regard, an early re-biopsy should be considered if targeted biopsy of an mG3 lesion yields no or low-grade PCA (ISUP 1) given the high prevalence of higher grade PCA in this group. Patients with mG1 lesions (also ≥ 1.5 cm) may receive primarily follow-up mpMRI if they qualify for active surveillance. Therefore, it might increase early detection of higher grade PCA and on the other hand, it might reduce re-biopsy rates in patients with ISUP 1 PCA within mG1 and thus increase AS safeness. For lesions in mG2, imaging is not unequivocal and may be impaired, for example due to coexistent signs of prostatitis. Follow-up mpMRI instead of prompt re-biopsy may be justified if the clinical setting allows.

Some limitations of this study, besides the retrospective design, need to be discussed. First, the definition of ADC value thresholds for the MRI grading groups was based on the literature and clinical- and scanner-specific experience [15, 31] The assessed values seem reasonable in our circumstances and clinical settings. Nevertheless, the values may be subjective to a certain extent, so that different thresholds may be defined at other institutions. Therefore, before using MRI grading groups, it has to be verified that the definitions are suitable for the individual settings. Second, the defined parameters offer a good aggregation of PCA characteristics and should be considered and evaluated in image analysis. However, there is no guarantee for completeness and more parameters may have an influence on MRI grading. Third, even if the examined parameters correlated with the different ISUP groups, a definite distinction is not possible merely based on this information, so that other (clinical) factors always need to be taken into account. Further research is required to evaluate performance and accuracy of MRI grading between non-significant and significant prostate cancer in detail. This means differentiation between ISUP groups 1–2 and ISUP groups 3–5 as conducted for ADC before [32]. Fourth, cancer detection on T2w sequences is qualitatively conducted in a subjective way, differentiating between discreet and clear lesions. As there is no quantitative approach established, this probably leads to low interreader and intrareader variability. Finally, we have no radical prostatectomy results or follow-up data included to determine the final patient outcome. It might be interesting to prove MRI grading in a prospective study design.

In conclusion, our study indicates that several mpMRI parameters correlate with the biopsy ISUP grade groups. Combination of these parameters using defined MRI grading groups (mG1 to mG3) seems to be helpful in addition to the standard PI-RADS classification for the better prediction of PCA aggressiveness and may offer more certainty for clinicians in further clinical pathway and their individual treatment selection or monitoring. Moreover, our data reveal that in patients within mG3 and no or low-grade PCA detection after biopsy an early re-biopsy should be considered, due to the high prevalence of higher grade PCA. Patients with mG1 lesions may receive follow-up mpMRI first if they qualify for active surveillance.

## Supplementary Information

Below is the link to the electronic supplementary material.

Supplementary file1 (DOCX 357 KB)

## Abbreviations

ADC: Apparent diffusion coefficient
DCE: Dynamic contrast enhancement
DWI: Diffusion-weighted imaging
EPE: Extraprostatic extension
EPI: Echoplanar imaging
IL: Index lesion
ISUP: International Society of Urological Pathology
LMM: Linear mixed model
mpMRI: Multiparametric magnetic resonance imaging
PCA: Prostate cancer
PIRADS: Prostate Imaging and Reporting Archiving Data System
PSA: Prostate-specific antigen
PSAD: Prostate-specific antigen density
PZ: Peripheral zone
ROI: Region of interest
TZ: Transition zone

## Declarations

### Guarantor

The scientific guarantor of this publication is Lars Schimmöller.

### Statistics and biometry

One of the authors has significant statistical expertise.

### Methodology

• Retrospective

• Diagnostic and prognostic study

• Performed at one institution
