# Supplementary material for: MRI grading for the prediction of prostate cancer aggressiveness
Source: Eur Radiol. 2021 Nov 8;32(4):2351–9. doi: 10.1007/s00330-021-08332-8 (PMC8921105; doi:10.1007/s00330-021-08332-8)

**Supp. Table 1:** Linear mixed model with ADC values of ss-EPI DWI to evaluate prediction of the ISUP grade group.

| n=125 | | **B** | **95%-CI** | **p** |
| --- | --- | --- | --- | --- |
| **MRI** | **Fixed Term** | 2.86 | 1.88 – 3.85 | **<0.001** |
|  | **Cross-zonal growth** | -0.28 | -0.81 – 0.26 | 0.206 |
|  | **T3 stage** | -0.44 | -1.16 – 0.28 | 0.228 |
|  | **Signal increase on high b-value images** | -0.06 | -0.49 – 0.61 | 0.2 |
|  | **ADC value (ss-EPI)** | -0.0012 | -0.00025 - 0.0026 | 0.104 |
|  | **MRI grading group** | 1 = -1.5  2 = -0.5  3 = Reference | -2.5 – -0.7  -1.1 – 0.9  Reference | **<0.001** |

*PCA = prostate cancer; DWI = diffusion weighted imaging; ADC = apparent diffusion coefficient;*

*B = estimation; CI = 95%-confidence-interval; p = p-value; ss-EPI = single slice echoplanar imaging;*

**Supp. Table 2:** Acquisition parameters for Magnetom Prisma® Siemens

|  | **Sequences** | | | | |
| --- | --- | --- | --- | --- | --- |
|  | T2 TSE | T2 TSE | T2 TSE | Resolve DWI | DCE dynamic T1 Vibe |
| **orientation** | Sagittal | Coronal | Axial | Axial | Axial |
| **TR (ms)** | 4060 | 3880 | 3990 | 5450 | 3.87 |
| **TE (ms)** | 101 | 99 | 102 | 50/80 | 1.46 |
| **Matrix size** | 320 | 320 | 256 | 140 | 256 |
| **Voxel size (mm)** | 0.5x0.5x3 | 0.5x0.5x3 | 0.5x0.5x3 | 1.4x1.4x3 | 0.8x0.8x3.3 |
| **FOV** | 170 | 170 | 130 | 200 | 200 |
| **b-values (sec/mm²)** | - | - | - | 0, 1000, 1800 (calc.) | - |
| **Acquisition time (min)** | 3:23 | 4:16 | 5:19 | 6:38 | 3:06 |

*TSE = turbo spin echo; DWI = diffusion weighted imaging; DCE = dynamic contrast enhancement; Vibe = volumetric interpolated breath-hold examination; TR = time of repetition; TE = time of echo; FOV = field of view*

**Supp. Table 3:** Acquisition parameters for Magnetom Skyra® Siemens

|  | **Sequences** | | | | |
| --- | --- | --- | --- | --- | --- |
|  | T2 TSE | T2 TSE | T2 TSE | Resolve DWI | DCE dynamic T1 Vibe |
| **orientation** | Sagital | Coronal | Axial | Axial | Axial |
| **TR (ms)** | 7870 | 7430 | 8770 | 6200 | 3.9 |
| **TE (ms)** | 98 | 98 | 79 | 56/84 | 1.39 |
| **Matrix size** | 320 | 320 | 256 | 112 | 256 |
| **Voxel size (mm)** | 0.5x0.5x3 | 0.5x0.5x3 | 0.5x0.5x3 | 1.8x1.8x3 | 0.8x0.8x3.3 |
| **FOV** | 170 | 170 | 130 | 200 | 200 |
| **b-values (sec/mm²)** | - | - | - | 0, 1000, 1600 (calc.) | - |
| **Acquisition time (min)** | 2:45 | 3:06 | 4:58 | 7:33 | 3:02 |

*TSE = turbo spin echo; DWI = diffusion weighted imaging; DCE = dynamic contrast enhancement; Vibe = volumetric interpolated breath-hold examination; TR = time of repetition; TE = time of echo; FOV = field of view*

**Supp. Table 4:** Acquisition parameters for Magnetom Trio® Siemens

|  | **Sequences** | | | | |
| --- | --- | --- | --- | --- | --- |
|  | T2 TSE | T2 TSE | T2 TSE | EPI DWI  (ss-EPI) | DCE dynamic T1 Vibe |
| **orientation** | Sagital | Coronal | Axial | Axial | Axial |
| **TR (ms)** | 11330 | 11330 | 10630 | 4700 | 3.62 |
| **TE (ms)** | 103 | 103 | 117 | 90 | 1.27 |
| **Matrix size** | 256 | 256 | 256 | 136 | 128 |
| **Voxel size (mm)** | 0.7x0.7x3 | 0.7x0.7x3 | 0.7x0.7x3 | 1.5x1.5x3 | 1.5x1.5x3.3 |
| **FOV** | 170 | 170 | 128 | 200 | 192 |
| **b-values (sec/mm²)** | - | - | - | 0, 500, 1000, 1400 (calc.) | - |
| **Acquisition time (min)** | 4:11 | 4:11 | 8:21 | 4:31 | 3:36 |

*TSE = turbo spin echo; DWI = diffusion weighted imaging; DCE = dynamic contrast enhancement; Vibe = volumetric interpolated breath-hold examination; TR = time of repetition; TE = time of echo; FOV = field of view*

**Sup. Fig. 1:** Point Plots for correlation of ISUP grade group with PSA, Age, ADC value (rs-EPI) and PCA diameter.


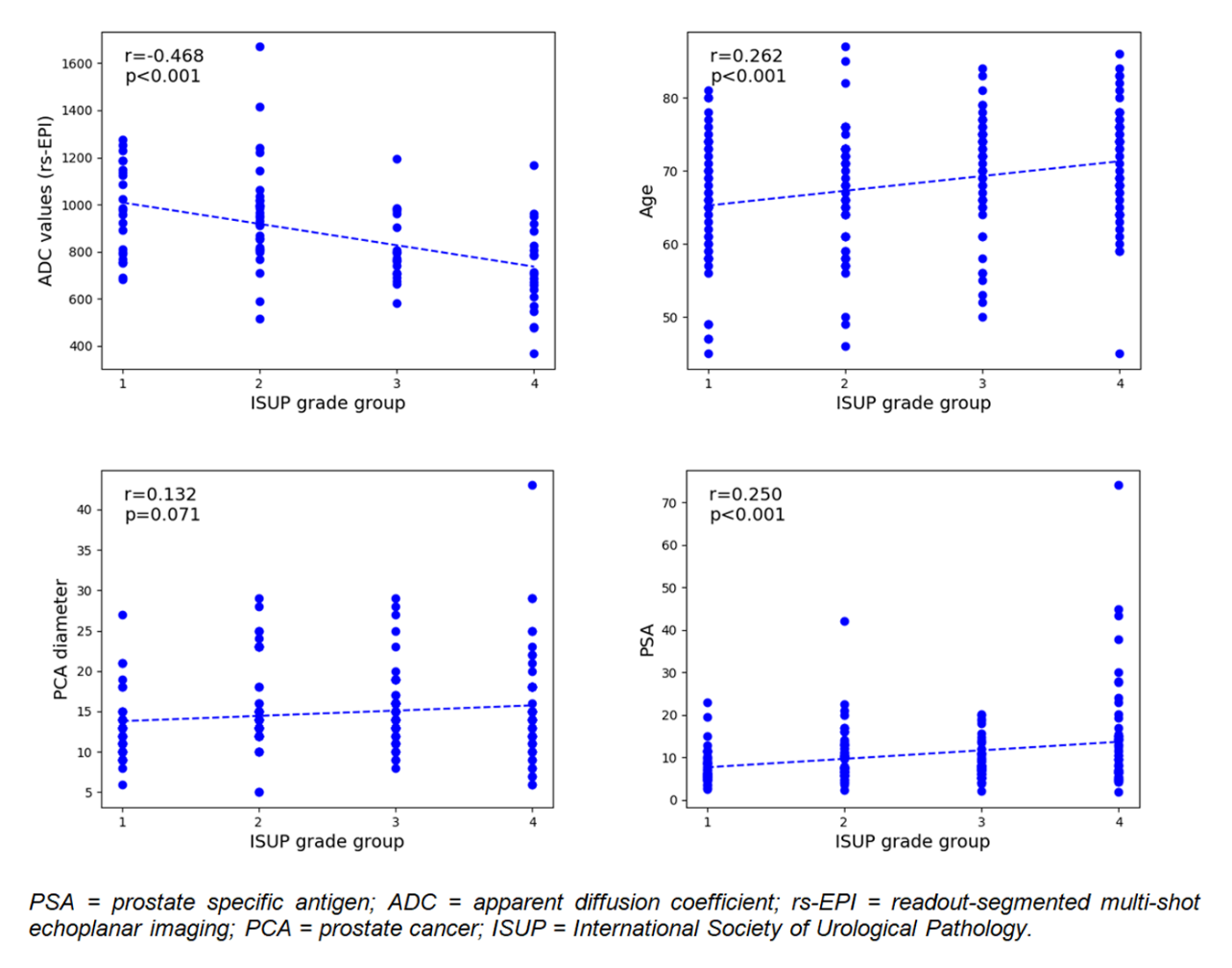

Supplement: Supplementary file 1 — Supplementary file1 (DOCX 357 KB) [file 330_2021_8332_MOESM1_ESM.docx]
